# Application of an abstract concept across magnitude dimensions by fish

**DOI:** 10.1038/s41598-020-74037-5

**Published:** 2020-10-09

**Authors:** Maria Elena Miletto Petrazzini, Caroline H. Brennan

**Affiliations:** 1 Department of Biomedical Sciences, University of Padua, 35131 Padua, Italy; 2 School of Biological and Chemical Sciences, Queen Mary University of London, London, E1 4NS UK

**Keywords:** Psychology, Zoology

## Abstract

Mastering relational concepts and applying them to different contexts presupposes abstraction capacities and implies a high level of cognitive sophistication. One way to investigate extrapolative abilities is to assess cross-dimensional application of an abstract relational magnitude rule to new domains. Here we show that angelfish initially trained to choose either the shorter of two lines in a spatial task (line-length discrimination task) or the array with “fewer” items (numerical discrimination task) spontaneously transferred the learnt rule to novel stimuli belonging to the previously unseen dimension demonstrating knowledge of the abstract concept of “smaller”. Our finding challenges the idea that the ability to master abstract magnitude concepts across domains is unique to humans and suggests that the circuits involved in rule learning and magnitude processing might be evolutionary conserved.

## Introduction

Concepts, the mental representations of classes of things, are the foundation of human knowledge. The ability to conceptualise promotes cognitive economy by reducing the need to learn each new stimulus encountered in everyday life and allows quick and adaptive responses^1–3^. Relational concepts are based on the relationship between stimuli and not on their physical resemblance, consistent with abstraction abilities^3,4^. Relational knowledge is indispensable to adapt and survive in complex and continuously changing environments and plays a key role in higher cognitive processes. The capacity to elaborate relational concepts and apply them to different contexts has long been considered a hallmark of human cognition controlled by the prefrontal cortex^5^. This belief has been challenged by studies showing that animals can learn relational concepts such as “same/different”, “above/below” or “larger than/smaller than” within a single domain^3,4,6,7^. There is also evidence that honeybees are able to transfer the concept of same-different from the olfactory domain to the visual domain^8^ and can also make a transfer from discrete (number) to continuous (size) magnitudes^9^.

The ability to discriminate between quantities is crucial to increase individual’s fitness (e.g., reduction of predation risk, adjustment of mating strategies)^10^. It is widely acknowledged that humans share with other animals an approximate non-symbolic system for representing number (Approximate Number System) in which accuracy is affected by the ratio between the quantities to be compared. This ratio-dependence signature characterizes a variety of magnitude judgements (e.g., length, size, duration) thus suggesting the existence of a common system for representation of both discrete (i.e., number) and continuous quantities (i.e., space, time) in both humans and non-human animals; the so-called “A Theory Of Magnitude” (ATOM)^11–14^. Although this theory is still controversial^15^, an ability to transfer a concept across magnitude domains would support the existence of a common system involved in quantity discrimination. However, spontaneous cross-dimensional transfer of a magnitude rule without specific training in animals is largely unexplored.

Fish are appealing to address this issue as recent studies have shown they possess cognitive skills previously considered to be a prerogative of mammalian brains. For instance, some species transmit cultural information, display episodic-like memory, possess numerical skills and use tools^16–19^. Here, we asked whether angelfish (*Pterophyllum scalare*) can spontaneously transfer a relational magnitude rule acquired along one dimension (numerical) to another one (spatial) and vice versa thereby testing both their abstraction abilities and the existence of a common magnitude system. Half of the fish were trained to select the shorter of two lines with a 0.5 length ratio, the other half were trained to select the array containing “fewer” dots in 10 vs. 20 comparison (0.5 numerical ratio). Numerical stimuli were controlled for non-numerical cues (i.e., cumulative surface area, density and convex hull) to avoid the possibility of using the perceptual properties of the numerical stimuli to learn the discrimination (See “Materials and Methods” below for more information). Once fish mastered the task, they were tested with stimuli belonging to the other dimension (fish trained with numbers were tested with lines and vice versa) to assess their ability to extrapolate the learnt rule to novel stimuli outside of the initial training domain.

## Results

**Figure 1:**
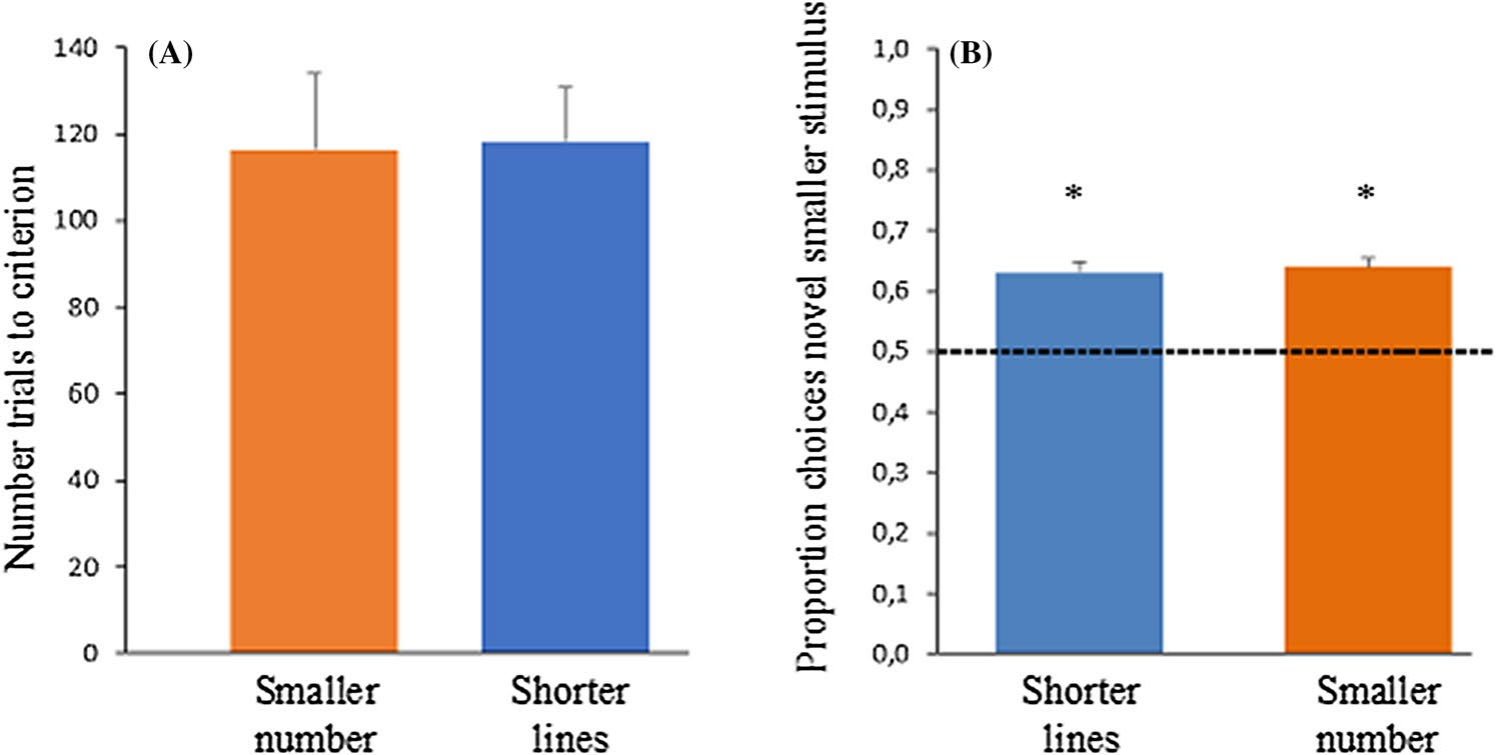
Results of the training and the test phase: (**A**) Number of trials to reach the criterion during the training phase for fish trained on dots (orange) and fish trained on lines (blue). (**B**) Proportion of choices for the novel smaller stimulus in the test phase. Fish trained on numbers significantly selected the shorter lines (blue) and fish trained on lines significantly chose the set with fewer dots (orange). Dashed line at 0.5 represents chance level performance. Data are represented as mean ± SEM. Significance from chance level performance is indicated by *P < 0.05.

The fish did not show a spontaneous preference for either the smaller number of dots (mean ± st. dev: 0.451 ± 0.083; one-sample Wilcoxon signed rank test: Z = 1.414, p = 0.157) or the shorter line (0.405 ± 0.131; Z = 1.633, p = 0.102) during the first training session. Mean trials to learning criterion was 117.43 ± 38.86, with no difference between the fish trained on numbers and those trained on lines (independent t-test: t(12) = 0.079, p = 0.938, Fig. 1A) showing that the level of difficulty was comparable between the tasks. There was no difference in the proportion of correct choices on the basis of the cumulative surface area control (congruent: 0.623 ± 0.055; incongruent: 0.640 ± 0.047; equated: 0.669 ± 0.047; repeated measures ANOVA: F(2, 12) = 1.368, p = 0.292), or convex hull and density (respectively: 0.661 ± 0.060; 0.628 ± 0.085; paired t-test: t(6) = 0.619, p = 0.558). Correct choices were significantly above chance in all stimulus controls (one-sample t-test: congruent: t(6) = 5.899, p = 0.001; incongruent: t(6) = 7.910, p < 0.001; equated: t(6) = 9.477, p < 0.001; convex hull: t(6) = 7.126, p < 0.001; density: t(6) = 3.995, p = 0.007), thus showing that under conditions in which discrimination could not be based on non-numerical cues, fish were using numerical information.

During the test phase, fish trained on number exhibited a significant preference for the shorter line (one-sample t-test: t(6) = 7.862, p < 0.001) and the fish trained on lines significantly chose the smaller number of dots (one-sample t-test: t(6) = 8.041, p < 0.001), with no difference between the two groups (independent t-test: t(12) = 0.283, p = 0.782, Fig. 1B).

## Discussion

Over the past decades, the field of comparative cognition has dramatically grown and thrived as an area of inquiry in biological and social sciences to understand the evolution of the human mind^20^. In particular, abstract thinking, relational knowledge and numerical competence have long been the center-piece of theories of human intelligence as they represent higher cognitive functions primarily attributed to the prefrontal cortex^21,22^. The ability to make rule-based decisions on magnitude relations across dimensions is fundamental in technology and science. However, non-verbal precursors of this ability are poorly investigated, and its evolutionary origin is almost unknown.

Cross-dimensional transfer of relational concepts requires two levels of information processing. The first involves encoding the relationships between the stimuli beyond their physical attributes, and the second implies the ability to extrapolate and transpose the abstract rule to a novel context. Our results indicate that angelfish formed the concept of “smaller” as they spontaneously transferred the learnt rule (choose the smaller one) to the novel stimuli belonging to the previously unseen dimension thus suggesting a coherent representation of magnitude across contexts. Furthermore, the use of multiple pairs of training stimuli and the presentation of completely novel visual stimuli in the test phase excluded the possibility of simple stimulus generalisation and provide strong evidence of abstract concept learning^4,6,23^.

This study shows for the first time that fish are capable of spontaneously applying an abstract relational concept across dimensions despite lacking a defined neocortex. This is important as abstraction and conceptualisation of magnitude across dimensions have long been thought to be a uniquely human capability^5^ that is already displayed at birth before the acquisition of language^24^. However, recent studies showed that other cognitive skills, traditionally ascribed to the mammalian neocortex, are supported by different neural circuitry in animals lacking a neocortex^25^. For instance, volitional orienting, which is linked to neocortical regions in humans, has been described in archerfish^26^. Here we propose that a functionally homologous circuit underlying relational magnitude conceptualisation may exist in non-mammalian species. This hypothesis is supported by our finding that magnitude representation across dimensions was symmetrical (i.e., same performance irrespective of the trained dimension). The spontaneous ability to create mappings across different dimensions indicates that the fish brain is predisposed to treat number and space as related. These results are in line with the existence of a common magnitude system to process quantitative information, as proposed by ATOM, which would not be necessarily cortically dependent in animals as recently suggested in birds^14^. Thus, complex magnitude extrapolative capabilities may not be uniquely human, but rather may extend to other species with relatively simple neural systems.

However, an alternative explanation may account for our results. In fact, although ATOM has been one of the most prominent theories in recent decades, an increasing number of studies providing behavioural and neural evidence of inconsistencies in spatial, temporal and number representations suggests that distinct modules are responsible for processing quantities^27–31^. According to this hypothesis, it would not be necessary to invoke a unique magnitude system to explain our findings. Rather, each dimension (number and length) would have been processed separately by different systems and only the concept of ‘smaller’ was abstract enough to be applied to both dimensions thus providing similar behavioural patterns in the two tasks. However, the debate about the existence of a common system and whether it is evolutionarily pervasive is still an object of discussion^15^ with future studies being necessary to understand how quantities are represented more broadly.

Lastly, despite controlling the stimuli for the non-numerical variables (cumulative surface area, density and convex hull) usually controlled for in numerical cognition studies in animals^32–38^, one may argue that the fish used contour length as a discriminative cue, as previously suggested in infants^39^. In this scenario, fish choice of the smaller stimulus in the test phase would have been the result of a generalisation process based on contour length rather than the extrapolation of a concept from one dimension to another. However, several studies on both humans and animals have shown that contour length is not a relevant cue to discriminate quantities^40–46^. For instance, adult humans cannot make a discrimination between two arrays of multiple items differing in quantity based on contour length alone^47^. Similar results have been reported in fish^48^. Further, with respect to this issue, it has been argued that the presentation of multiple items can hinder the ability to represent continuous quantities, such as contour length, as the mechanisms for keeping track of distinct individuals would be overwhelmed^40,49,50^. However, we cannot formally exclude the possibility that the fish were at least partially using contour length as the discriminative cue in our study. Further experimentation controlling for this perceptual cue is needed before drawing any firm conclusion about the exact strategy adopted by fish.

## Materials and methods

### Subjects

Subjects were 20 juvenile angelfish obtained from a local commercial supplier. We used the same sample size previously used to study numerical abilities in angelfish with the same protocol here described^35^. Fish were stocked at the Laboratory of Comparative Psychology (University of Padua) for at least 15 days before the beginning of the experiment. The subjects were maintained in groups of 6–8 individuals in 150-l aquaria provided with an air filter and enriched with natural vegetation and gravel. Water temperature was kept at a constant temperature of 26 ± 1 °C and a 12:12 h light: dark (L:D) photoperiod with an 18-W fluorescent light. Before the experiment, fish were fed twice daily with commercial food flakes (GVG-Mix).

All husbandry and experimental procedures complied with the national guidelines and regulations for the care and use of animals of the country (Italy, Decreto Legislativo 4 Marzo 2014, n. 26) in which they were performed. The procedures employed were approved by the Scientific Committee on Animal Health and Animal Welfare (Organismo Preposto al Benessere Animale, OPBA) of the University of Padova (Protocol n.31/2015).

### Apparatus and stimuli

**Figure 2:**
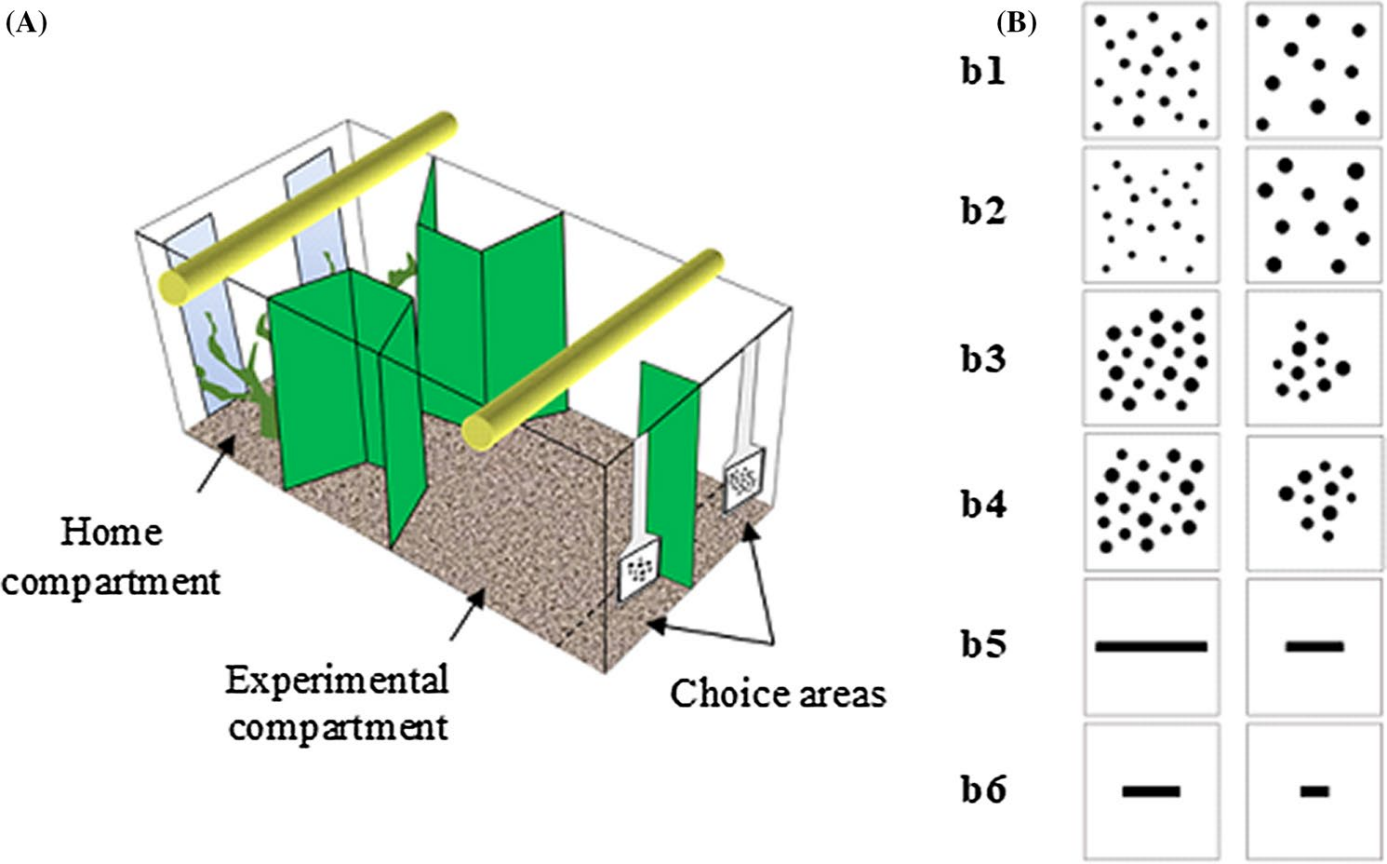
Experimental apparatus and stimuli. (**A**) The apparatus was divided into a home compartment and an experimental compartment connected by a corridor. Stimuli were presented in the front experimental compartment where the choice areas were delimited by an opaque divider. Example of a numerosity discrimination trial. (**B**) Schematic representation of the stimuli used. Example of numerical stimuli with cumulative surface area equal (b1), incongruent (b2) and congruent (b3, b4). In (b1) and (b2) stimuli are controlled for convex hull and in (b3, b4) for density. Stimulus (b4) is a rotated version of (b3). Example of line stimuli in which the same length could be either the shorter (b5) or the longer line (b6) within the pairs across trials.

The experimental apparatus consisted of a glass tank (60 × 40 × 35 cm) filled with gravel and 30 cm of water maintained at a temperature of 26 ± 1 °C and was provided with two air filters. Green plastic partitions were used to divide the tank into a front “experimental compartment” and a back “home compartment” connected by a 10 × 8 cm corridor (Fig. 2A). The home compartment (15 × 40 cm) was provided with natural vegetation and two mirrors (29 × 6 cm) placed on the short wall in order to reduce the potential effects of social isolation^51^. Two 18-W fluorescent lamps illuminated the apparatus, one for each compartment. The area adjacent to the front wall of the experimental apparatus was divided into two identical choice areas (6 × 20 cm) by a green plastic divider (30 × 6 cm). The exterior walls of the tank were covered with green plastic material except the home compartment and the front wall where a green net acted as one-way screen to prevent the fish from being influenced by external visual stimuli.

During the pre-training phase, the stimulus consisted of a single white square (6 × 6 cm), whereas during the training and the test phase we used two sets of stimuli: line stimuli and numerical stimuli. Line stimuli consisted of pairs of horizontal black lines of different lengths (range 10–54 mm) on a white background (6 × 6 cm). Stimuli were extracted from a pool of 18 different pairs and the length ratio within each pair was always equal to 0.5 (e.g., 24 vs 48 mm length). Furthermore, some lengths could be either the shorter or the larger line within the pairs across trials (e.g., 12 vs. 24 mm or 24 vs. 48 mm, Fig. 2B). Numerical stimuli consisted of pairs of black dots on a white background (6 × 6 cm). We used a single numerical comparison: 10 vs. 20 dots with a 0.5 numerical ratio. Here we used a single numerical comparison rather than multiple contrasts as fish have been previously found to spontaneously learn a relative quantitative rule based on the relation between the stimuli presented (e.g., select the array with more/less items) rather than an absolute rule (e.g., select the array with a specific number of items), even when they are trained with a single contrast^35,52^. Since this issue has not been investigated in length discrimination tasks in fish, we used multiple line comparisons to guarantee learning of a relational concept. Numerical stimuli were extracted from a pool of 24 pairs of stimuli. However, since stimuli could be rotated on each of their side, each stimulus had four different possible orientations, for a total of 96 different pattern configurations (Fig. 2B). To prevent the fish from learning the discrimination based on the overall configuration, the size of the dots (diameter range 2.7–7 mm) and their spatial distribution varied across sets. Non-numerical cues that could co-vary with numerosity (i.e., density, cumulative surface area, convex hull) were varied among trials^32–38^. In detail, in one third of the stimuli the cumulative surface area (i.e., summation of the areas of all items in each stimulus) was equal for the two numerosities. In another third the cumulative surface area was incongruent with the number of dots (i.e., the larger numerosity had the smaller cumulative surface area) and in the remaining third the cumulative surface area was congruent with the number of dots (i.e., the smaller numerosity had the smaller cumulative surface area). Furthermore, half of the stimuli were controlled for the convex hull (i.e., the overall space encompassed by the most lateral figures was the same), whereas the other half was controlled for density (i.e., the inter-item distance within each pair was the same) (Fig. 2B). Since fish proved to be unable to use perimeter as a cue in quantity discrimination tasks^48^, we controlled for the continuous variables most frequently used in numerical cognition studies in animals, fish included^32–38^. The fish were trained to select either the shorter line or the set with fewer items as previous studies showed no difference in accuracy between fish trained toward the larger stimulus as positive and those trained toward the smaller stimulus as positive^36,37,52–54^. In the test phase, the fish previously trained on the number task, were presented in probe trials with the line stimuli and the fish trained on the line-length task were tested with the numerical stimuli in which the cumulative surface area was matched. The stimuli were presented at the bottom of two transparent plastic supports that were externally hung on the front wall of the tank.

### Procedure

We used a procedure previously adopted to investigate the numerical abilities in angelfish^35^. Four days before the beginning of the experiment, fish were individually housed in the experimental tank in order to familiarize them with the environment. During these days, fish were fed twice daily with commercial food flakes released in the centre of the experimental compartment by using a Pasteur pipette. The experimental procedure consisted of three different phases: pre-training, training and test phase.

### Pre-training

During the pretraining, the fish underwent 2 sessions of 6 trials each (one in the morning and one in the afternoon) for a total of 12 trials per day, for 5 days a week. Inter-session interval lasted 4 h. Each trial started with the subject in the home compartment with a rectangular panel made with green net (36 × 8 cm) placed into the corridor and a green opaque barrier placed in front of it to prevent sight of the stimuli during setup of the trial. The stimulus (a white square) was hung outside the tank and the left/right position was counterbalanced within each session, with the stimulus not being presented on the same side more than 2 consecutive times in a row, to avoid the development of side bias. The opaque barrier was then removed to allow the subject to look at the stimulus through the net. In order to familiarize the fish with the procedure, the green net was removed as soon as the subject entered the corridor to allow it access to the experimental compartment. Entering the correct choice area led to a food reward (a mixture of water and food flakes) provided in close association with the stimulus with a Pasteur pipette. The stimulus was removed only after the subject had finished eating and was outside the choice area. The subject was then gently ushered into the home compartment using a transparent plastic panel to proceed to the next trial. Once the subject had approached the white square in 10 out of 12 trials (83%) in one day the pretraining was concluded. Training started the following day.

### Training

We used a procedure similar to the one described in the pretraining but here the subjects were presented with two stimuli and they were allowed to look at them for 10 s before removing the net panel from the corridor. Fish were randomly assigned to one of two conditions: half of the fish (N = 10) were trained on the numerical discrimination task whereas the other half (N = 10) was trained on the line-length discrimination task. In the numerical task, all fish were trained to choose the smaller numerosity in a 10 vs. 20 comparison. The left/right position of the stimuli was counterbalanced over trials. If the fish entered the choice area in correspondence to the correct stimulus (10), it received the food reward. If the fish chose the wrong stimulus, it was immediately ushered toward the home compartment in order to start a new trial. There was no time limit for a response to occur. In the line-length task, all fish were trained to choose the shorter of the two lines. Correct and incorrect choices led to the same consequences as described for the numerical training. After reaching the learning criterion of 75% (9 out of 12) correct trials over two consecutive days, the fish started the test phase. As dependent variables, we determined the number of trials to reach the learning criterion and the proportion of choices for the correct stimulus. If the subject did not reach the criterion within 15 days, the training phase was interrupted and it did not start the test phase. Six fish were excluded from the experiment at this stage.

### Test

Fish underwent 6 probe trials intermingled with 8 standard trials for 4 consecutive days (14 daily trials divided into two sessions of 7 trials each) for a total of 24 probe trials and 32 standard trials. In probe trials, the fish previously trained to select the smaller numerosity in the numerical discrimination task were presented with the line stimuli and the fish trained to select the shorter line in the line-length discrimination task were presented with 10 versus 20 dots. During the probe trials, the stimuli were removed as soon as the fish made a choice irrespective of the response and no food reward was provided (extinction procedure). In the standard trials, the fish were presented with the same stimuli used during the training phase and were normally rewarded with food^35–37,52,53^. In this phase, we computed the proportion of choices for the novel smaller stimulus presented (i.e., the shorter line for fish trained on numbers and the smaller numerosity for fish trained on lines).

### Statistical analysis

During the training phase, the number of trials required to reach the learning criterion was compared between the fish trained on the numerical task and those trained on the line-length task by using an independent t-test. To evaluate whether fish had a spontaneous preference for the smaller training stimulus, the proportion of correct choices in the first training session was analysed by means of one-sample Wilcoxon signed rank test to compare the performance to chance level (50%). To determine whether there was any difference in accuracy among the stimuli controls, a repeated measure ANOVA was used with area control as factor of analysis and a paired t-test was used to compare performance between convex hull and density controls. One-sample t-tests were used to assess whether the proportion of correct choices for each stimulus control was different from a random value of 50%. To evaluate whether fish transferred the learned rule to the new dimension in the test phase, the proportion of choices for the novel smaller stimulus was analysed by means of one-sample t-tests. An independent t-test was used to compare test performance of the two groups.

All statistical tests were two tailed, α was set up at 0.05. Non-parametric and parametric statistics were used based on normality distribution of data verified by Shapiro–Wilk tests. Data were analysed using SPSS 25.0.
